# Phylogenetic Analysis of Guinea 2014 EBOV Ebolavirus Outbreak

**DOI:** 10.1371/currents.outbreaks.84eefe5ce43ec9dc0bf0670f7b8b417d

**Published:** 2014-05-02

**Authors:** Gytis Dudas, Andrew Rambaut

**Affiliations:** Institute of Evolutionary Biology, University of Edinburgh, Edinburgh, UK; University of Edinburgh, Edinburgh, UK

**Keywords:** disease outbreak, ebolavirus, Guinea, zoonoses

## Abstract

Members of the genus Ebolavirus have caused outbreaks of haemorrhagic fever in humans in Africa. The most recent outbreak in Guinea, which began in February of 2014, is still ongoing. Recently published analyses of sequences from this outbreak suggest that the outbreak in Guinea is caused by a divergent lineage of Zaire ebolavirus. We report evidence that points to the same Zaire ebolavirus lineage that has previously caused outbreaks in the Democratic Republic of Congo, the Republic of Congo and Gabon as the culprit behind the outbreak in Guinea.

## Introduction

A recent article^1^ suggests that the currently ongoing outbreak in Guinea is caused by a divergent variant of the Zaire ebola (EBOV) lineage. The EBOV strain has previously caused ebola outbreaks in the Democratic Republic of Congo (DRC), the Republic of Congo (RC) and Gabon. The authors publish three complete genome sequences from the Guinea outbreak and perform a phylogenetic analysis using 24 sequences of the Zaire and other representative lineages. One finding is that the 2014 sequences fall as a divergent lineage outside the Zaire lineage suggesting that this may be a pre-existing endemic virus in West Africa rather than the result of spread of the EBOV lineage from the Central African countries that have had previous human outbreaks.

Previously, a dynamic re-interpretation of EBOV emergence in Central Africa has been suggested, citing correlations between time, geographic distance and genetic distance of Ebola haemorrhagic fever outbreaks^2^ and the recent ancestry of related EBOV lineages in fruit bats^3^.

## Materials and Methods

All complete genome sequences from the genus Ebolavirus (which includes Bundibugyo BDBV, Reston RESTV, Sudan SUDV, Tai Forest TAFV and Zaire ebolavirus EBOV species) were collated from genbank including the sequences from the Guinea outbreak. Genbank accessions and sources for the sequences can be found at http://epidemic.bio.ed.ac.uk/ebolavirus_sequences.

The Ebolavirus genome consists of a single strand of negative sense RNA and contains 7 protein coding genes (in order 3'-NP-VP35-VP40-GP-VP30-VP24-L, separated by various intergenic regions)^4^. We collated the protein coding regions of each gene (alignment length 14647 nucleotides) and, in a separate alignment, the non-coding intergenic regions. Phylogenetic trees were inferred in PhyML^5^ or MrBayes^6^ using the GTR^7^+Γ substitution model. We were able to replicate the analysis presented in Baize *et al*.^1^ only when omitting the accommodation of rate heterogeneity modelled as a discretized Γ distribution. We suspect the difficulty in replicating the analysis is due to a combination of using different sequences, a different alignment and the inherently unreliable rooting of the EBOV clade using highly divergent sequences from other ebolavirus clades. We have uploaded the alignments we used (whole genome, coding and non-coding) to a GitHub repository at https://github.com/evogytis/ebolaGuinea2014.

We also compiled a dataset containing only the glycoprotein (GP) sequences, for which more sequences are available. Many of the extra sequences come from wild ape carcasses^8^ in Gabon and RC.

These sequences were analyzed in BEAST^9^ to establish a time frame for the split of the Guinea viruses from other EBOV lineages. The data were analyzed using the GTR+Γ nucleotide substitution model, an uncorrelated relaxed molecular clock (following a lognormal distribution)^10^ and under different demographic models (constant population size, exponential growth or the non-parametric Bayesian skyride^11^).

GP sequence results were recovered from a relaxed molecular clock analysis, under an exponential growth tree prior (as it can accommodate a constant population size scenario when the growth rate is 0) but the analysis was found to be quite robust to different demographic models.

## Analysis

An alignment of complete genomes and a maximum likelihood tree (PhyML) appears to confirm the phylogenetic position shown in the recent paper^1^ (Figure 1), albeit the position of the Guinea outbreak sequences is not very well supported.

**ML tree of complete genomes.**
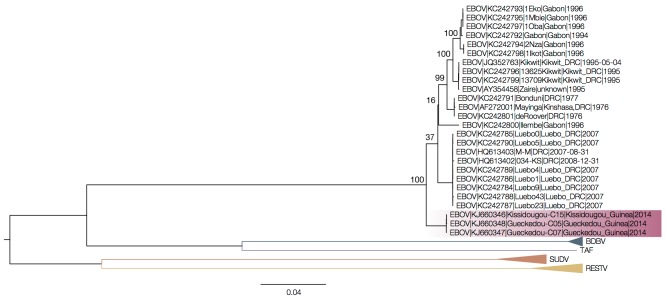
ML tree of complete genomes without accommodating for rate heterogeneity shows the Guinea outbreak sequences (highlighted) as belonging to a divergent EBOV lineage. Tips belonging to the EBOV lineage are not collapsed. Numbers above key nodes in the EBOV clade are bootstrap values (100 replicates).

When the intergenic sequences are removed, however, the Guinea outbreak sequences fall within the diversity of Zaire ebolavirus (Figure 2).

**MrBayes tree of concatenated coding sequences.**
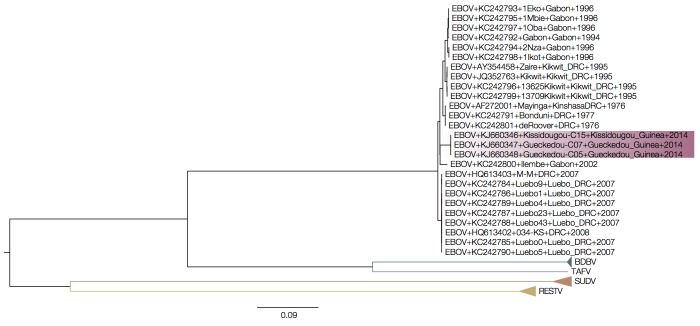
When only the coding sequences are used, the Guinea outbreak sequences appear to be derived from within the diversity of Gabon/DRC EBOV lineages.

**Expanded view of a MrBayes tree of concatenated coding sequences.**
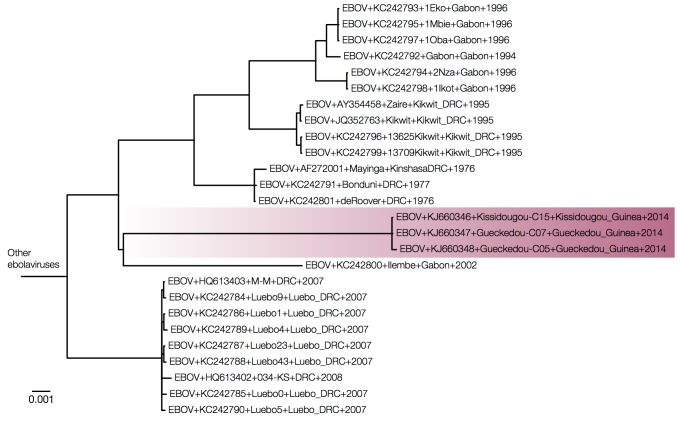
Expanding the EBOV region of the tree (same tree as Figure 2, but with the divergent ebolavirus species cropped out) we see that the Guinea outbreak sequences are nested within the EBOV clade.

**MrBayes tree of intergenic sequences.**
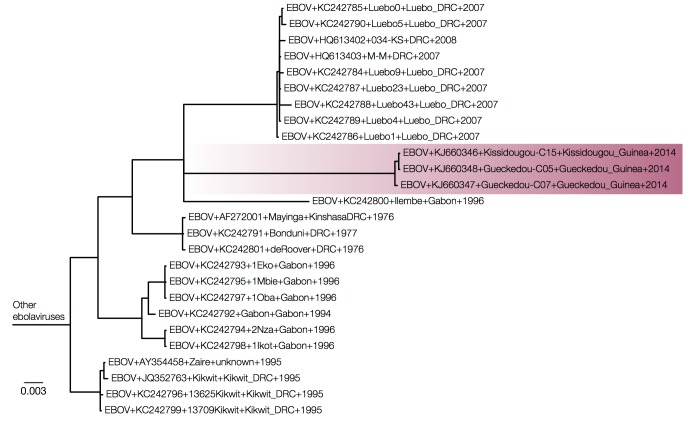
Intergenic regions show a similar picture with the Guinea sequences nested within EBOV.

EBOV lineages are rather poorly sampled and sequences from most outbreaks, because of the nature of the outbreaks, have nearly identical sequences. The branch leading to the Guinea outbreak is long, not because it is a divergent lineage but because it is the most recently sampled so has had the most time to evolve. Combined with a very divergent outgroup this leads to a situation where the root position of the EBOV clade is unreliably estimated.

Figures 3 and 4 show MrBayes trees from protein coding and intergenic regions of the EBOV genome, respectively, with more divergent ebolavirus strains cropped out. Note that trees in Figures 3 and 4 are essentially identical but differ by where the other ebolavirus species root the EBOV clade (on the 2007 Gabon outbreak for the coding regions in Figure 3 and on the 1995 Kikwit outbreak for the intergenic regions in Figure 4). This shows that the rooting of this clade using the highly divergent other ebolavirus species is very problematic.

However, EBOV is estimated to evolve at about 7×10^-4^ substitutions per site per year^12^ which means that the virus will accumulate significant amounts of substitutions over the nearly 40 years since the first recorded outbreak in 1976. We can use this to root the EBOV tree and look at where the Guinea outbreak lies. Path-O-Gen (available at http://tree.bio.ed.ac.uk/software/pathogen/) was used to find the root that gave the best association between genetic divergence and time.

The relationship between genetic divergence and time after rooting the tree using least squares regression is shown in Figure 5.

**Root-to-tip regression of a MrBayes tree of concatenated coding regions.**
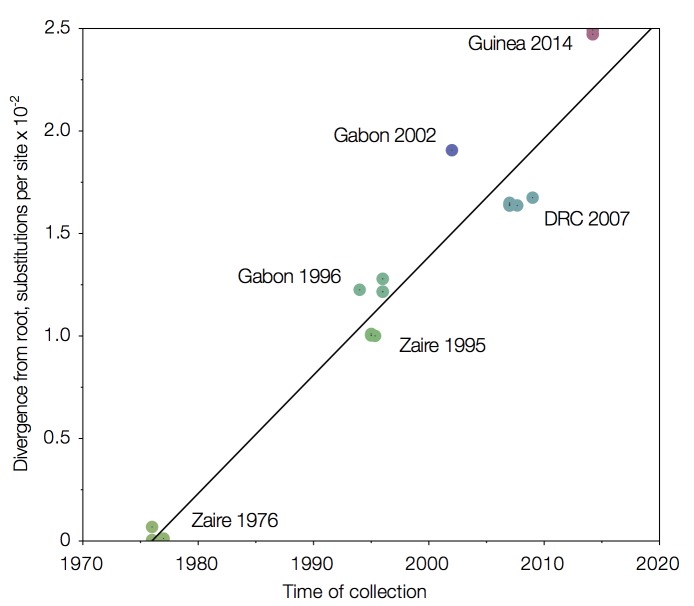
Sequences from the 1976 Zaire outbreak are very close to the root.

**MrBayes tree of concatenated coding sequences rooted by least squares regression.**
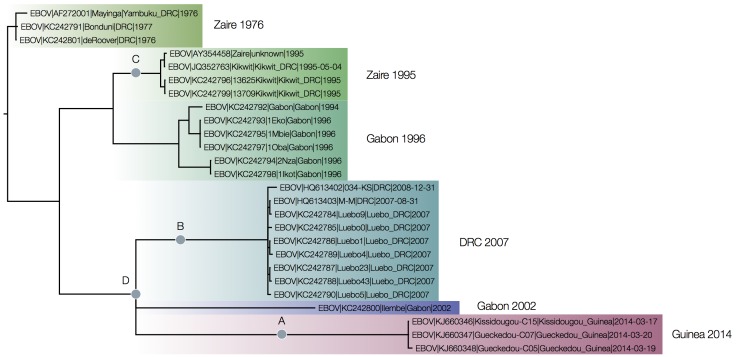
The Bayesian posterior support for all the groupings between the outbreaks are 1.0 including for the grouping of Guinea 2014 with DRC 2007 and Gabon 2002. This demonstrates that the uncertainty about the position of the Guinea 2014 lineage in the complete ebolavirus trees was down to the rooting of the EBOV clade (*i.e.*, where the divergent outgroups connect to the EBOV tree). The relationships of the EBOV outbreaks is completely consistent for the simple whole genome alignment, the coding regions only and the intergenic regions only but the position of the root changes. In the figure A) denotes the position of the root for the full genome maximum likelihood tree, B) for the Bayesian coding-sequence only tree, C) the Bayesian intergenic regions only tree and D) the combined coding-sequence and intergenic region accommodating different rates of evolution.

Figure 6 shows the phylogeny of the coding sequences recovered by MrBayes (a maximum likelihood tree using PhyML gave an almost identical tree) rooted by least squares regression. The root of this tree is very close to the earliest sequences from the 1976 Zaire outbreak.

## Estimating the date of introduction of EBOV into Guinea

The analysis of GP sequences in BEAST revealed rooting consistent with that found in Figure 6 as well as a nucleotide substitution rate (mean of lognormal distribution from which the rates were drawn is 1.07×10^-3 ^substitutions per site per year, 95% HPD interval 5.99×10^-4^ - 1.75×10^-3^) on a scale expected, given previously published rates^12^ and the fact that GP codes for a surface glycoprotein.

In Figure 7 the estimate of the split between the lineage now causing an outbreak in Guinea and the Central African lineage that had caused outbreaks in DRC and Gabon is late 2002 (95% HPD interval 2000 - 2006). This gives us a lower boundary on the introduction of Central African lineage of EBOV into Guinea, although these estimates should be interpreted with caution. We also find very good support for the common ancestry of Guinea and DRC/Gabon lineages (posterior probability = 1.0).

Figure 7 also highlights the importance of environmental sampling - many sequences in the tree come from ape carcasses and are more diverse (not shown) than sequences from human outbreaks, giving this dataset much better resolution.

**Maximum clade credibility tree of GP sequences.**
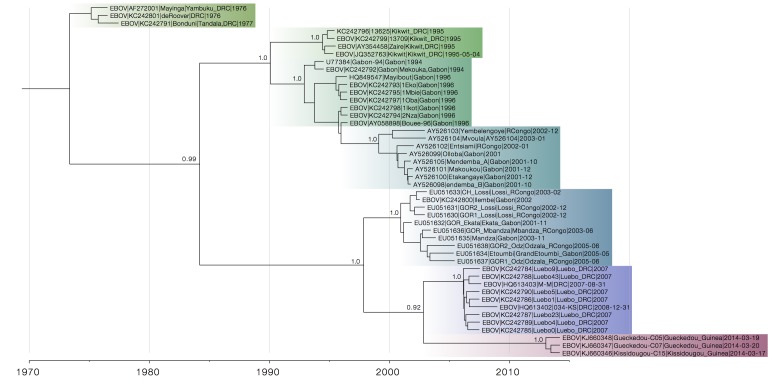
Although the closest relatives of the Guinea lineage are not entirely certain (posterior probability 0.92), its relationship with Central African EBOV lineages is well-supported (posterior probability 1.0).

## Conclusion

The phylogenetic analysis of the five ebolavirus species here does not substantially improve on that presented by Baize *et al*.^1^ in that even when partitioning the alignment into coding and non-coding regions we get inconsistent rooting positions for the EBOV clade. We believe that at present no suitable outgroup sequences to root the EBOV phylogeny exist and that a temporal rooting gives the most consistent results.

This approach indicates that the outbreak in Guinea is likely caused by a Zaire ebolavirus lineage that has spread from Central Africa into Guinea and West Africa in recent decades, and does not represent the emergence of a divergent and endemic virus.

As the GP sequences show, without more diverse sequences, especially those from the animal reservoir, it is difficult to narrow down the estimates of when and through what means the Central African EBOV lineage has been introduced into West Africa.

## Competing Interests

The authors have declared that no competing interests exist.
